# Qualitative and Quantitative Analysis of B-Cell-Produced Antibodies in Vitreous Humor of Type 2 Diabetic Patients with Diabetic Retinopathy

**DOI:** 10.1155/2020/4631290

**Published:** 2020-06-30

**Authors:** Baoyi Liu, Yijun Hu, Qiaowei Wu, Yunkao Zeng, Yu Xiao, Xiaomin Zeng, Ying Fang, Liang Zhang, Tao Li, Honghua Yu

**Affiliations:** ^1^Guangdong Eye Institute, Department of Ophthalmology, Guangdong Provincial People's Hospital, Guangdong Academy of Medical Sciences/The Second School of Clinical Medicine, Southern Medical University, Guangzhou, China; ^2^Aier Institute of Refractive Surgery, Refractive Surgery Center, Guangzhou Aier Eye Hospital, Guangzhou, China; ^3^Aier School of Ophthalmology, Central South University, Changsha, China; ^4^State Key Laboratory of Ophthalmology, Zhongshan Ophthalmic Center, Sun Yat-sen University, Guangzhou, China

## Abstract

**Aim:**

To analyze the levels of B-cell-produced antibodies in the vitreous humor of patients with or without diabetic retinopathy (DR) both qualitatively and quantitatively.

**Methods:**

A total of 52 type 2 diabetes mellitus (T2DM) with DR patients and 52 control subjects without diabetes mellitus or inflammatory diseases were included in this prospective study. The levels of immunoglobulin (Ig)A, IgM, and IgG subtypes were measured using a magnetic color-bead-based multiplex assay.

**Results:**

The concentrations of IgA, IgM, and total antibodies in the DR group were significantly higher than those in the control group (all *p* < 0.001), but there was no significant difference in the 4 IgG subtypes between the two groups after Bonferroni correction. Pearson's correlation analysis revealed low negative correlations between levels of antibodies (IgA, IgM) and estimated glomerular filtration rate (eGFR, *r* = −0.443, *r* = −0.377, respectively, both *p* < 0.05). Furthermore, multiple linear regression analysis yielded three equations to predict the concentrations of IgA, IgM, and total antibodies in the vitreous humor according to eGFR and other clinical variables (*r* = 0.542, *r* = 0.461, and *r* = 0.312, respectively, all *p* < 0.05).

**Conclusion:**

Increased levels of IgA, IgM, and total antibodies produced by B cells were observed in the vitreous humor of T2DM patients with DR. There were low negative correlations between levels of antibodies (IgA, IgM) and eGFR.

## 1. Introduction

Diabetes mellitus (DM) is a chronic metabolic disorder that is characterized by hyperglycemia, resulting in insulin resistance. According to the latest statistics, there are 463 million people currently with DM in the world, and this continues to rise [1]. Type 2 diabetes mellitus (T2DM) is the most common form of DM, accounting for 91% of DM. Hyperglycemia control reduces the mortality and microvascular complications associated with the disease [2, 3]. Diabetic retinopathy (DR) is one of the important microvascular complications of DM and is the leading cause of blindness in DM population. Inflammation is regarded as a critical component in the pathogenesis of DR [4, 5]. The clinical findings in patients with DR include (1) increased levels of inflammatory biomarkers such as vascular endothelial growth factor (VEGF) and C-reactive protein in the serum [6]; (2) increased levels of inflammatory cytokines and chemokines such as tumor necrosis factor-alpha, interleukin- (IL-) 1, IL-6, and C-C motif ligand (CCL) 3 in the aqueous and vitreous humor [7, 8]; and (3) detection of inflammatory cells such as neutrophils, macrophages, and lymphocytes in the proliferative epiretinal membrane of DR patients [9]. These in turn confirm the contribution of inflammatory factors in the pathogenesis of DR.

B cells play key roles in the production of cytokines and antibodies in humans and mice [10, 11] and were found to regulate inflammation in patients with DM [12–15]. Antigen-specific antibodies that are produced by activated B cells are the first-line defense against pathogens in exposed surfaces, and this is done by neutralizing antigens, facilitating phagocytosis and antigen presentation [16]. Besides, the self-reactive antibodies are involved in the destruction of self-tissues and initiation of autoimmune diseases [17]. Thus, B-cell-mediated immune response and regulation are important in immune response, and these B-cell functions might also contribute to the development of DR. However, there is limited evidence on the activation of B cells in DR patients.

In the current study, the concentrations of B-cell-produced immunoglobulin (Ig)A, IgM, and IgG subtypes in vitreous humor of T2DM patients with DR and control subjects were analyzed. Furthermore, the correlations between the concentrations of these antibodies and clinical variables of DR were investigated.

## 2. Materials and Methods

**Figure 1:**
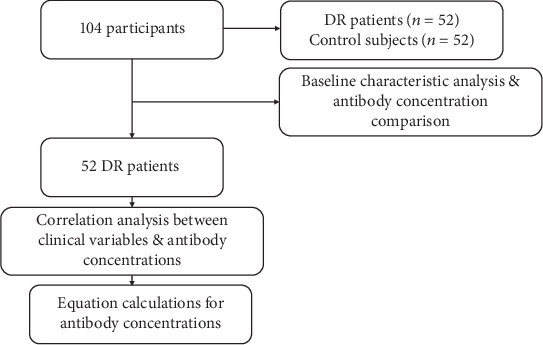
Flow chart of the study selection process. Abbreviation: DR: diabetic retinopathy.

This prospective study was conducted from May 2018 to March 2020 in accordance with the tenets of the Declaration of Helsinki. This study obtained ethical approval from the local Research Ethics Committee of the Guangdong Provincial People's Hospital (Number: 2016232A) before conducting the study. Informed consent was obtained from all patients. A flow chart of included population and analyses is shown in Figure 1. T2DM was diagnosed by endocrinologists based on the diagnostic criteria of the American Diabetes Association [18]. Diagnosis and classification of DR were confirmed according to the international clinical diabetic retinopathy severity scales [19]. Patients who underwent vitrectomy for vitreous hemorrhage, proliferative epiretinal membrane, or tractional retinal detachment were included. The control group included patients without DM but underwent vitrectomy for idiopathic preretinal membranes, idiopathic macular holes, or rhegmatogenous retinal detachment. The primary endpoint of the study was follow-up at one month after vitrectomy surgery. The patients were regularly followed up after that. The exclusion criteria were as follows: patients (1) with other ocular conditions associated with inflammation (such as age-related macular degeneration, glaucoma, and uveitis), (2) with a history of ocular surgery or trauma, (3) who received anti-VEGF treatment, and (4) with a history of severe systemic inflammatory diseases, primary kidney diseases, or any other kidney diseases that are the cause other than DM secondarily. All subjects underwent a complete ocular examination and blood pressure, fasting blood glucose (FBG), glycated hemoglobin (HbA1c), serum creatinine (sCr), blood urea nitrogen (BUN), estimated glomerular filtration rate (eGFR), and urinary albumin to creatinine ratio (UACR) which were measured before surgery. The value of eGFR was calculated based on the Chronic Kidney Disease Epidemiology Collaboration (CKD-EPI) equation according to the guidance of an experienced nephrologist (Levey [20]). All patients underwent pars plana vitrectomy in accordance with the standardized operation procedures using the 23-gauge trocar and cannula system (Alcon Laboratories, Inc. Fort Worth, Tex. the USA). About 0.2-0.4 ml of vitreous humor was aspirated into a sterile syringe before intraocular infusion. The vitreous samples were centrifuged immediately at 2500 rpm at 4°C for 10 min. The supernatants were aspirated and subsequently stored at -80°C until further analysis.

The Bio-Plex Pro™ Human Isotyping Panel, 6-plex kit (#171A3100M, control 64190954, Bio-Rad Laboratories, Inc., Hercules, CA, USA) was used to measure the concentrations of 6 human antibodies, including IgA, IgM, IgG1, IgG2, IgG3, and IgG4. The experimental procedures were conducted according to the manufacturer's instructions. Next, 40 *μ*l of undiluted vitreous humor sample was used for the reaction and finally analyzed the fluorescence intensity from the immunoassay using the Bio-Plex™ 200 System (software version 6.1, Bio-Plex Manager, Bio-Rad Laboratories).

### 2.1. Statistical Methods

Statistical analyses were performed using IBM SPSS statistics version 19.0 (IBM SPSS Statistics; IBM Corporation, Chicago, IL, USA). One-way ANOVA was performed for evaluating the sex differences between the DR group and the control group. Data normality was confirmed by Shapiro-Wilk test. Independent, two-tailed Student's *t*-tests were performed to compare other clinical variables and concentrations of the antibodies between the two groups. Bonferroni-corrected significance threshold (*p* = 0.006) was used for the multiplicity of measurement of antibodies between the two groups. Pearson's correlation test was used to analyze the associations between the clinical variables and the concentrations of antibodies. Furthermore, multiple linear regression analysis was used to yield equations for calculating the concentrations of antibodies according to the clinical variables that are statistically significant in Pearson's correlation analysis. Sample size calculation was performed by using a web-based simple power/sample size calculation, UCSF Biostatistics: Power and Sample Size Programs, https://www.stat.ubc.ca/~rollin/stats/ssize/, *α* = 0.006 (after Bonferroni correction), power = 0.90, and two-sided test. A two-tailed *p* < 0.05 was considered to be statistically significant.

## 3. Results

### 3.1. Baseline Characteristics of the Included Subjects

Fifty-two DR patients (10 eyes with vitreous hemorrhage, 24 eyes with proliferative epiretinal membrane, and 18 eyes with tractional retinal detachment according to the primary diagnosis) and 52 non-DR subjects (17 eyes with idiopathic preretinal membranes, 17 eyes with idiopathic macular holes, and 18 eyes with rhegmatogenous retinal detachment) were recruited, including 63 males and 41 females. Clinical characteristics of the DR and the control group are presented in Table 1. The levels of FBG, HbA1c, sCr, BUN, and UACR in the DR group were significantly increased (all *p* < 0.001), while eGFR was significantly decreased (*p* < 0.001) when compared to those in the control group. There were no significant differences in other clinical characteristics including age, gender, systolic blood pressure, and diastolic blood pressure between the two groups (*p* > 0.05 for all).

### 3.2. B-Cell-Produced Antibodies in the Vitreous Samples

**Figure 2:**
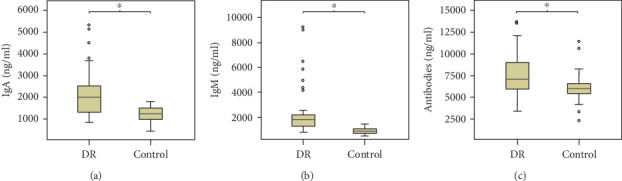
The concentrations of IgA, IgM, and antibodies in the vitreous humor of the two groups. Abbreviations: DR: diabetic retinopathy; Ig: immunoglobulin. (a) The concentration of IgA antibody (ng/ml) in the vitreous humor of the DR patients and the control group. (b) The concentration of IgM antibody (ng/ml) in the vitreous humor of the DR and control groups. (c) The concentration of antibodies (ng/ml) in the vitreous humor of the DR group and the control group. The levels of IgA, IgM, and total antibodies in the vitreous humor were significantly higher in the DR group than in the control group. ^∗^Statistically significant *p* value < 0.001 by independent, two-tailed Student's *t*-tests.

The concentrations of B-cell-produced antibodies between the DR and the control group are shown in Table 2. The concentrations of all the antibodies were within the detection limit, and the results revealed that IgA, IgM, and total antibodies in the DR group were significantly higher than those in the control group after Bonferroni correction (all *p* < 0.001). A detailed description on the levels of IgA, IgM, and total antibodies between the two groups was shown in Figure 2. The four IgG subtypes showed no significant differences between the two groups after Bonferroni correction (*p* > 0.006 for all).

### 3.3. Correlations

There were low correlations between clinical variables and levels of B-cell-produced antibodies (Table 3). The concentration of IgA was positively correlated with FBG (low correlation with a *r* = 0.317, *p* = 0.001) and negatively correlated with eGFR (low correlation with a *r* = −0.443, *p* < 0.001). The concentration of IgM was positively correlated with UACR (low correlation with a *r* = 0.363, *p* < 0.001) and negatively correlated with eGFR (low correlation with a *r* = −0.377, *p* < 0.001).

### 3.4. Calculating Equations for Antibodies in Vitreous Humor

Multiple linear regression analysis using clinical variables (such as the duration of DM and DR, eGFR, and UACR) was performed to predict IgA, IgM, and total antibody values, which yielded three equations:

IgA (ng/ml) = −15.805∗age (years) − 11.342∗eGFR (ml/min/1.73 m^2^), *r* = 0.542, *p* < 0.001, and standard error of estimate = 776.67 ng/ml.

IgM (ng/ml) = −10.861∗eGFR (ml/min/1.73 m^2^) + 0.447∗UACR (mg/g), *r* = 0.461, *p* < 0.001, and standard error of estimate = 1394.90 ng/ml.

Total antibodies (ng/ml) = 6749.95 − 14.473∗eGFR (ml/min/1.73 m^2^), *r* = 0.312, *p* = 0.016, and standard error of estimate = 2019.53 ng/ml.

These three equations showed that the concentrations of IgA, IgM, and total antibodies in the vitreous humor of the DR patients were associated with eGFR, which is a marker for kidney damage.

## 4. Discussion

Systemic inflammation is associated with the whole course of T2DM, and it plays an important role in the development and progression of DR. Activation of B cells contributed to the development of DM in recent years [21–23]. However, the immune response mediated by B cells has been rarely reported in the DR patients [24]. This study analyzed the B-cell-produced antibodies including IgA, IgM, and four IgG subtypes in the vitreous humor of T2DM patients with DR both qualitatively and quantitatively. The concentrations of IgA, IgM, and total antibodies were significantly increased in the DR group when compared to the control group. Besides, there were low negative correlations between levels of antibodies (IgA, IgM) and eGFR. These results might shed light on novel insights regarding the role of B cells in the development and progression of DR.

In the present study, the concentrations of IgA, IgM, and total antibodies, but none of the IgG subtypes, were increased in the vitreous humor of patients with DR. The reason for the increase of IgA might be due to this type of antibody being “spared” by the phagocytes in the eye, as it mainly possesses receptors for Fc fragments of other antibodies, e.g., IgG [24]. On the other hand, increase in IgM might be caused by the destruction of the blood vessel-retinal tissue barrier in DR and is followed by a large number of antigens and inflammatory cells entering the retina and vitreous humor to trigger an acute inflammatory response [25]. In the future, the above hypotheses and the functional roles of antibodies in the pathogenesis of DR require further investigation. With regard to the concentration of IgG, no significant difference between DR patients and control subjects was detected. These findings were similar to the results of a previous study [26]. Taken together, these results suggested that IgG might not play a significant role in the pathogenesis of DR. As known, the first Ig that is synthesized during the early phase of humoral immune response is IgM, which acts as a first-line defense. The humoral immune system switches to the production of IgG that serves as the subsequent defense and is responsible for immune memory. The increased levels of IgM in the DR patients of our study indicate that the development of DR might be associated with an acute ocular humoral immune response. However, similar IgG levels in DR patients and control subjects suggest that long-term immune memories of triggering factors might not be developed in eyes with DR.

Correlation analysis revealed low negative correlations between levels of antibodies (IgA, IgM) and eGFR, a marker of kidney damage. These negative correlations suggested that kidney damage tended to be more severe in patients with stronger B-cell-mediated immune responses. Moreover, multiple linear regression yielded three equations for predicting the concentrations of IgA, IgM, and total antibodies in the vitreous humor according to eGFR, suggesting possible associations between retinopathy and nephropathy during the development of DM [27–29]. The mechanisms of the associations between ocular B-cell-produced antibodies and eGFR in T2DM still remain unknown. Considering that diabetic nephropathy and diabetic retinopathy are microvascular complications of T2DM, a potential “common pathway” might exist in their underlying mechanisms. A previous study has demonstrated that persistent hyperglycemia and insulin resistance could lead to the progression of vascular inflammation and dysfunction of endothelial cells [30]. When this process occurs in kidneys, it causes glomerular filtration dysfunction, resulting in diabetic nephropathy [31]. When the similar process affects eyes, it led to progressive breakdown of hematoocular barrier and occurrence of DR [32]. If microvascular complications occur, then the activated B cells reach the damaged sites through the blood vessel wall and produce antibodies. In addition, the antibodies produced by the activated circular B cells can also be carried to the damaged sites by the blood flow. These pathophysiological processes might be the mechanisms that underlie the increased concentrations of antibodies in the vitreous humor of T2DM patients with DR, although further studies are warranted to figure out the exact mechanisms. Moreover, based on the above results with regard to the correlation analysis and multiple linear regression analysis, the extent of ocular B-cell activation in the vitreous humor of DR patients showed correlation with the levels of certain serum metrics. These findings remind us of the necessity of cooperation of ophthalmologists with endocrinologists and renal physicians to monitor microvascular damage in DM [33, 34].

The present study for the first time reported that local humoral immune response was involved in the pathogenesis of DR in T2DM, suggesting that the B cells might play an important role in the development and progression of DR. However, there were several limitations that should be acknowledged in the current study. Firstly, the sample size is not large and the study is not randomized which may lead to selection bias. Although significant differences have been observed using the included number of samples, population-based randomized studies with large sample sizes are needed to validate the results of the present study. Secondly, it would be more convincing if a group of diabetic patients without DR was added. However, during patient recruitment, very few T2DM patients without DR who met the inclusion criteria were referred to our clinic. Thirdly, the concentrations of antibodies in the serum samples were not analyzed. Although the previous studies have proven that the levels of plasma antibodies were increased in DR [14, 35], it would be better if the serum antibodies were measured in our patients.

## 5. Conclusions

This study showed that higher levels of IgA, IgM, and total antibodies produced by B cells were detected in the vitreous humor of T2DM patients with DR. There were low negative correlations between concentrations of IgA and IgM in the vitreous humor and eGFR, indicating a potential relationship between retinopathy and nephropathy in T2DM. Further investigations are needed to verify the functional roles of B cells in DR and other microvascular complications of diabetes.

## Figures and Tables

**Table 1:** Clinical characteristics of the subjects.

	DR (*n* = 52)	Non-DR (*n* = 52)	*p* value
Age (y)	53.56 ± 12.05	57.17 ± 9.72	0.095
Male/female	30/22	33/19	0.552
Duration of DM (years)	7.37 ± 8.59	N/A	N/A
Duration of DR (months)	7.10 ± 9.79	N/A	N/A
SBP (mmHg)	129.19 ± 18.72	128.19 ± 18.29	0.783
DBP (mmHg)	77.90 ± 10.93	79.25 ± 10.47	0.523
FBG (mmol/l)	9.64 ± 3.44	6.30 ± 1.77	<0.001^∗^
HbA1c (%)	7.67 ± 1.53	5.99 ± 0.77	<0.001^∗^
sCr (*μ*mol/l)	256.52 ± 237.52	86.86 ± 49.28	<0.001^∗^
BUN (mmol/l)	12.37 ± 8.23	6.00 ± 2.64	<0.001^∗^
eGFR (ml/min/1.73 m^2^)	37.02 ± 22.51	85.42 ± 20.34	<0.001^∗^
UACR (mg/g)	903.69 ± 873.52	19.91 ± 17.13	<0.001^∗^

Abbreviation: DM: diabetes mellitus; DR: diabetic retinopathy; SBP: systolic blood pressure; DBP: diastolic blood pressure; FBG: fasting blood glucose; HbA1c: glycated hemoglobin; sCr: serum creatinine; BUN: blood urea nitrogen; eGFR: estimated glomerular filtration rate; UACR: urinary albumin to creatinine ratio. Duration of DM and DR were unavailable in the control group (*n* = 52). ^∗^*p* value of <0.001 by independent, two-tailed Student's *t*-tests was considered statistically significant. One-way ANOVA was used for evaluating the differences in sex.

**Table 2:** Concentrations of antibodies in the vitreous humor.

	DR (*n* = 52)	Non-DR (*n* = 52)	*p* value
IgG1 (ng/ml)	1654.05 ± 993.35	2196.35 ± 1310.44	0.019
IgG2 (ng/ml)	436.01 ± 236.54	529.7 ± 237.61	0.047
IgG3 (ng/ml)	478.6 ± 266.97	523.2 ± 212.83	0.348
IgG4 (ng/ml)	926.77 ± 888.32	1065.81 ± 589.86	0.350
IgG (ng/ml)	3495.42 ± 1682.07	4315.05 ± 1477.15	0.010
IgA (ng/ml)	2156.71 ± 1029.42	1376.49 ± 469.8	<0.001^∗^
IgM (ng/ml)	2013.55 ± 1877.88	471.28 ± 237.1	<0.001^∗^
Total antibodies (ng/ml)	7665.67 ± 2348.57	6162.82 ± 1480.91	<0.001^∗^

Abbreviation: DR: diabetic retinopathy; Ig: immunoglobulin. ^∗^Statistically significant by independent, two-tailed Student's *t*-tests (*p* value < 0.006 after Bonferroni correction).

**Table 3:** Correlations between clinical characteristics, serum metrics, and levels of antibodies.

Clinical characteristics	IgA (ng/ml)		IgM (ng/ml)		Antibodies (ng/ml)	
	*r* value	*p* value	*r* value	*p* value	*r* value	*p* value
Age (y)	-0.186	0.029^∗^	-0.123	0.108	-0.097	0.164
Sex	0.119	0.114	-0.082	0.204	-0.053	0.298
Duration of DM (years)	0.128	0.099	0.260	0.004^∗^	0.146	0.070
Duration of DR (months)	0.178	0.036^∗^	0.069	0.242	0.026	0.395
SBP (mmHg)	0.121	0.110	-0.027	0.392	-0.005	0.480
DBP (mmHg)	0.203	0.020^∗^	0.027	0.393	-0.008	0.468
FBG (mmol/l)	*0.317*	*0.001* ^∗^	0.132	0.091	-0.005	0.479
HbA1c (%)	0.296	0.001^∗^	0.276	0.002^∗^	0.201	0.020^∗^
sCr (*μ*mol/l)	0.276	0.002^∗^	0.147	0.068	0.094	0.171
BUN (mmol/l)	0.287	0.002^∗^	0.14	0.078	0.119	0.115
eGFR (ml/min/1.73 m^2^)	*-0.443*	*<0.001* ^∗∗^	*-0.377*	*<0.001* ^∗∗^	-0.289	0.001^∗^
UACR (mg/g)	0.110	0.133	*0.363*	*<0.001* ^∗∗^	0.185	0.030^∗^

Abbreviation: DM: diabetes mellitus; Ig: immunoglobulin; SBP: systolic blood pressure; DBP: diastolic blood pressure; FBG: fasting blood glucose; HbA1c: glycated hemoglobin; sCr: serum creatinine; BUN: blood urea nitrogen; eGFR: estimated glomerular filtration rate; UACR: urinary albumin to creatinine ratio. Italicized letters: low correlations were found between B-cell-produced antibodies and clinical variables of DM. ^∗^Statistically significant *p* value of <0.05 by Pearson's correlation analysis. ^∗∗^Statistically significant *p* value of <0.001 by Pearson's correlation analysis.

## Data Availability

The data used during the current study are available from the corresponding author on reasonable request.
